# T Cell Repertoire During Ontogeny and Characteristics in Inflammatory Disorders in Adults and Childhood

**DOI:** 10.3389/fimmu.2020.611573

**Published:** 2021-02-09

**Authors:** Svenja Foth, Sara Völkel, Daniel Bauersachs, Michael Zemlin, Chrysanthi Skevaki

**Affiliations:** ^1^ German Center for Lung Research (DZL), Institute of Laboratory Medicine, Universities of Giessen and Marburg Lung Center (UGMLC), Philipps University Marburg, Marburg, Germany; ^2^ Department of General Pediatrics and Neonatology, Saarland University Medical School, Homburg, Germany

**Keywords:** T cell immunity, diversity, clonality, ontogeny, autoimmune disorders, immune deficiencies, allergy, next generation sequencing

## Abstract

Since the first day of life, a newborn has to deal with various pathogens from the environment. While passive immune protection is provided by diaplacental maternal antibodies, the development of cellular immunity is ongoing. A mature immune system should be able not only to defend against pathogens, but should also be able to differentiate between self- and non-self-antigens. Dysregulation in the development of cellular immunity can lead to severe disorders like immunodeficiency, autoimmunity and chronic inflammation. In this review, we explain the role of T cell immunity in antigen detection and summarize the characteristics of a mature TCR repertoire as well as the current state of knowledge about the development of the TCR repertoire in ontogenesis. In addition, methods of assessments are outlined, with a focus on the advantages and disadvantages of advanced methods such as next generation sequencing. Subsequently, we provide an overview of various disorders occuring in early childhood like immunodeficiencies, autoimmunity, allergic diseases and chronic infections and outline known changes in the TCR repertoire. Finally, we summarize the latest findings and discuss current research gaps as well as potential future developments.

## Introduction: T Cell Immunity

### The Role of T Cell Receptor in Antigen Recognition

The cellular immune system is based on the interaction between T cells and antigen presenting cells. Short peptides, that are presented by MHC-I molecules on the cell surface of antigen-presenting cells, are detected by the T cell receptor (TCR) of T cells. The TCR is a heterodimeric receptor, composed of α- and β-chains or γ- and δ-chains. Each chain is composed of a constant region and a variable region, which is either composed of a V- and a J-region (α chain) or a V, a D and a J region (β chain). The variable domain contains the complementarity determining region (CDR3), which mediates antigen binding and is mainly responsible for TCR diversity and antigen specificity. While each T cell expresses its unique TCRs, a high degree of diversity of TCRs is necessary to detect a broad spectrum of foreign antigens. The TCR gene loci on Chromosome 7 for TRB and on chromosome 14 for TRA comprises over 250 different genes encoding for the V, D, and J region and diversity is provided by somatic gene rearrangement. Additionally, single nucleotides are inserted and deleted randomly (1).

Despite a broad repertoire to defend pathogens, the discrimination between self and non-self is crucial for immunocompetence. The selection of T lymphocytes takes place in the thymus and is known as central tolerance. Premature T lymphocytes emerging from bone marrow immigrate to the thymus to undergo a differentiation process including positive and negative selection. First, in the thymic cortex, only T cells with sufficient ability to bind to MHC molecules are not sorted out by apoptosis, which is called positive selection. During subsequent negative selection, T cells with high affinity to self-antigens are eliminated and only T cells without significant self-reactivity leave the thymus and immigrate to peripheral lymphoid organs (2).

Thus, the organism of a newborn is armed with a highly efficient cellular immune system, capable to defend itself against infections as well as tolerate self-antigens. Every disturbance in this highly regulated process can lead to inflammatory and autoimmune disorders or severe immunodeficiencies (1, 3, 4). Recent advances have shown that viral infections play a role in the central tolerance process and can lead to an impaired self-tolerance. For example, thymus atrophy occurs in acute virus infections and direct infiltration of the thymus epithelial cells by virus antigens is observed (5, 6). Thus, viral infections can influence antigen presentation and increase susceptibility to autoimmune disorders (7).

### Characteristics of the TCR Repertoire

#### Diversity and Clonality

**Figure 1:**
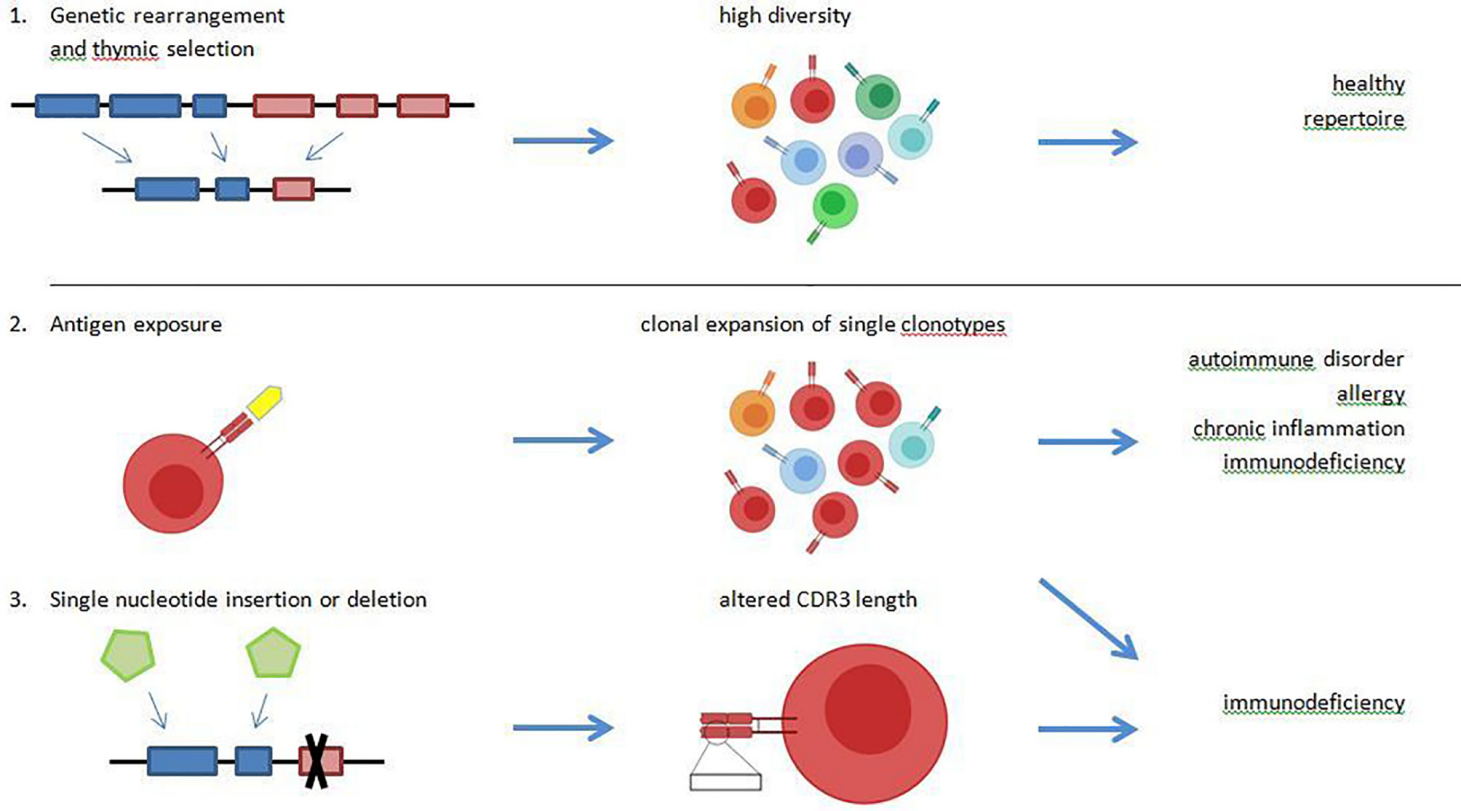
A healthy T cell receptor (TCR) repertoire is characterized by a high diversity and the ability to detect foreign antigens as well as tolerate self-antigens. These properties are mediated by genetic rearrangement of the VDJ-region, and positive and negative selection in the thymus (1.). Driven by antigen exposure, some TCR undergo clonal expansion, which leads to a reduced diversity of the overall repertoire. A high abundance of single clonotypes with reduced repertoire diversity is observed in chronic virus infections like Epstein-Barr virus (EBV) and cytomegalovirus (CMV), in allergy, in chronic inflammation and possibly in immunodeficiencies (2.). Diversity of the TCR repertoire may be mediated by random nucleotide insertion and deletion. In specific immunodeficiency disorders, a reduced CDR3-length is observed due to nucleotidedeletion (3.).

In a healthy immune repertoire, T lymphocytes that underwent the selection process in the thymus show a highly diverse receptor repertoire (polyclonal) and are thereby capable to defend almost against every pathogen (see **Figure 1**). Theoretically, the number of possible TCRß-chains is nearly limitless, but model calculations assume a number of 10^11^ possible ß-chains (8). New methodological approaches estimated an individual repertoire of about 10^6^ ß-chains (9). Once a suitable MHC-presented peptide is identified and binds to a TCR, the T cell changes to an effector cell and starts clonal expansion (see **Figure 1**). After a pathogen is eliminated, the count of clonal T cells declines and only a small amount of antigen specific T cells rest as memory cells in the circulating blood pool. These T cells can be reactivated in a second exposure to the same antigen with a higher and faster response. It is remarkable that the immune response shows significant differences between the primary and secondary antigen exposure. Bousso et al. could show that the selective expansion of specific T cells in the secondary response is independent from the relative frequency of memory T cell but may be associated to the epitope density (10). In chronic infections like EBV and CMV as well as in autoimmune diseases, a clonal expansion of T cells is observed and recent investigations showed that such TCR clones can serve as biomarkers (3, 11–13).

#### Private and Public TCR Clones

Considering the high diversity of an individual TCR repertoire, the statistical probability of finding the same clones in different individuals is rather low. Nevertheless, specific TCR clones are shared among individuals in a higher number than the statistical probability and are defined as “public”. It has been demonstrated that the quantity of public clonotypes is around 1% and that these TCRs are less dependent on inherited factors like the HLA alleles but influenced by antigen-exposure (9). On the other hand, some TCR clones are highly individual and thus called “private”. In some studies, public TCR clones are associated with autoimmune disorders. For example, a high amount of public TCR clones could be found in cerebrospinal fluid of patients with multiple sclerosis (14). In HIV-infected individuals, a high number of public TCRs is linked with a protective immune response (15, 16).

### Use of Next Generation Sequencing (NGS) and Data Analysis

Due to the enormous diversity the characterization of the TCR repertoire is a challenging task. Over the past decades, various methods have been developed to investigate the TCR repertoire. Each method may decipher a smaller or larger part of the repertoire. Several reviews provide a broad overview of the available methods, their advantages and disadvantages (see **Table 1**) (1, 26, 28). The increasing use of next generation sequencing (NGS) techniques in this field has provided new and high-resolution insights into the TCR repertoire of healthy and diseased individuals. NGS offers several advantages for diagnostic applications. Compared to other methods the resolution of information is much higher using less input of biomaterial. However, several comparative studies have shown that the different approaches lead to significantly different results, even when using the same input material (27, 30). These studies indicate that there is still a need to standardize the analysis of TCR repertoires for clinical application. This is not only the requirement of a patient-specific TCR sequencing but also the customizable data analysis and evaluation. This point in particular is currently being addressed by many scientists. In addition to the development of different analysis tools for NGS data, different analytical approaches are compared in order to satisfy the great diversity of the TCR repertoire. Different approaches are summarized and compared by Miho and colleagues as well as by Bradley and Thomas (31, 32). In the following years, these further developments will increase the understanding of the diversity and evolution of the TCR repertoire and allow T cell receptors to become even more important in clinical diagnostics than they are today.

**Table 1:** Methods of assessment.

**Methods**	**Biomaterial**	**Assessment**	**Advantages**	**Disadvantages**	**References**
**Flow cytometry**	T cells	Detection of expressed TCRs by monoclonal antibodies	* rapid screening of CD4+ and CD8+ T cells * no purification of cell populations necessary	* limited availability of monoclonal antibodies * high amount of cells is necessary * low-frequency T cell clones are not detected	Faint et al. (17) Cossarizza et al. (18) Flores-Conzalez et al. (19)
**CDR 3 spectratyping**	T cell DNA or RNA	Electrophoresis of amplicons derived from the CDR3 region	* rapid analysis of T cell clones with different length * semi-quantitative	* blind for the underlying sequence * low-frequency T cell clones are not detected	Cochet et al. (20) Currier et al. (21) Fozza et al. (22)
**Classical cloning combined with Sanger sequencing**	T cell DNA or RNA	Sequencing of amplicons derived from the CDR3 region	* In comparison to classical spectratyping it additionally reveals the DNA sequence	* large workload * low-frequency T cell clones are not detected * massive data sets to analyse	Sanger et al. (23) Sant’Angelo et al. (24) Correia-Neves et al. (25)
**NGS**	T cell DNA or RNA	Amplification of the CDR3 region followed by sequencing	* low amount of starting material DNA * highly abundant * proportional to -the number of T cells RNA * only expressed TCRs are analysed * less biased by PCR artefacts and efficiencies	* in a bulk assay the pairing information of α and β chain is absent * massive data sets to analyse DNA * primer efficiency affects the results RNA * not proportional to the number of T cells	Woodworth et al. (1) Six et al. (26) Liu et al. (27) Rosati et al. (28) Simone et al. (29) Barennes et al. (30)

### Development of TCR Repertoire

Although random events do play a significant role during the rearrangement of the genes of antigen receptors, the development of the BCR and TCR repertoires is strictly regulated during ontogeny and during the establishment of lymphocyte subpopulations (33, 34). In humans, fetal prothymocytes with rearranged TCR genes occur as early as 7 weeks of gestation in the yolk sac and liver. In the fetal blood, naïve CD38^+^CD45RA^+^ T cells predominate and the CD4/CD8 ratio is elevated in comparison to adults. Studies of human fetal tissues and cord blood revealed that the maturation of the T cell repertoire is mainly characterized by an expansion of clonality and by an increasing addition of N nucleotides within the hypervariable CDR3 region (35–37). Interestingly, the entire gene loci of the TCR alpha, beta, gamma and delta chains are accessible in the second trimester fetus, and the use of variable, diversity and joining gene elements appears to undergo fewer changes during ontogeny when compared to the immunoglobulin heavy chain genes (38). Overall, TCR repertoires are functionally more similar in cord blood but diverge during later life, paralleling the exposure to extrauterine antigens (37).

Schelonka et al. observed that TCR transcripts were clonally restricted in the second trimester fetus and were polyclonal later in life. The length of CDR3 regions increased during the fetal development in TCR transcripts regardless of their V region usage, due to an increasing addition of N nucleotides (35). γδ-T cells bearing a TCR with the Vγ9 and Vδ2 variable region (Vγ9Vδ2 T Cells) are typically reactive to phosphoantigens and predominate both among fetal and adult circulating T cells (36, 39). However, fetal Vγ9Vδ2 T cells express divergent CDR3 repertoires. Taken together with functional differences, this indicates that in contrast to murine γδ-T cells, the human adult Vγ9Vδ2 do not arise from the abundant fetal Vγ9Vδ2 T cell population but from the small number of Vγ9Vδ2 T cells generated in the postnatal thymus (39). Moreover, Ben Youssed et al. found that Vα7.2^+^ CD161high mucosal-associated invariant T (MAIT) cells which are reactive to microbial riboflavin precursor derivatives, acquire a memory phenotype within a few weeks of life depending on their antigen specificity. Thus, during the antigen-driven T cell memory formation, fetal T cell populations with other reactivities are diluted out over a period of at least 6 years (40).

## TCR Repertoire in Inflammatory Disorders

### Asthma and Allergy

Bronchial asthma is a chronic inflammatory disease and affects over 330 million people worldwide (41). It is one of the most common chronic disorders in childhood with up to 10% of children in Western Europe being afflicted (42). The pathogenesis of allergic asthma is characterized by activation of a TH2-immune response with the secretion of proinflammatory cytokines like IL-4 and IL-5, which in turn leads to an increase in IgE production as well as activation of mast cells and eosinophils (43). There is some evidence that allergic asthma is associated with a skewed TCR repertoire. In 1998, Hodges showed that the TRBV5-5/5-6 subset of the CD4+ T cell population was increased in asthmatics compared to healthy controls, albeit there was a high inter-individual difference in specific TCR frequency in both groups (44). Wahlström also demonstrated an altered TCBV usage in patients with allergic asthma with similar results for peripheral blood and bronchoalveolar lavage (45).

Likewise, in regard to atopy, Kircher et al. (46) found an increased frequency of certain Vß and Vα-chains in individuals with house dust mite allergy. Sade and colleagues (47) could show that the usage of Vß subsets is altered by specific immunotherapy (SIT). Recently, Cao et al. demonstrated a higher diversity and increased clonality in the TCR ß repertoire of allergic children compared to healthy controls using NGS methods (48). Such findings support the hypothesis, that antigen exposure in early life alters immune responses and leads to atopy and related disorders like bronchial asthma.

### Autoimmune Disorders

Autoimmune disorders are a heterogenic group of diseases, characterized by an altered immune response of auto-antibodies and self-reactive T cells that leads to inflammation and tissue damage in different organ systems. Studies based on methods like flow-cytometry and CDR3 spectratyping as well as recent research in NGS techniques demonstrate a decreased diversity of the TCR repertoire, as well as an increased number of public T cell clones in autoimmune disorders like systemic lupus erythematosus (SLE), arthritis and Crohn’s Disease (12, 49, 50), but these findings are not consistent For example, an animal model of autoimmune encephalomyelitis revealed that a reduced diversity of the TCRαβ repertoire is responsible for protection from autoimmunity (51). It is discussed that exposure to certain antigens leads to an increased susceptibility to autoreactivity. For example, in Myasthenia Gravis (MG) and in Typ1 diabetes, chronic viral infections with EBV and Coxsackievirus could be identified as a trigger for autoimmune responses (52–54). These findings of a skewed TCR repertoire with a clonal pattern in TCRß spectratyping could be confirmed by other authors with some evidence towards a more pronounced effect in systemic autoimmune disorders like SLE or juvenile polyarthritis compared to organ-specific disorders such as diabetes mellitus (49, 55).

So far, studies using high throughput sequencing methods in children are rare. Dokai et al. found a preferential use of Vß families in children with MG in the development and relapse phase, but not in the remission phase, supporting the hypothesis of antigen-driven selection of T cell clones (56). Eugster et al. used NGS in children with autoantibodies (e.g., pre-diabetes), revealing a highly diverse repertoire (57). This may be due to early investigation before the onset of disease and such findings should be reevaluated in other autoimmune disorders in children.

### Immunodeficiency

Primary immunodeficiencies are a group of rare diseases that leads to severe infections and often to a shortened lifespan. Susceptibility to bacterial infections is mainly mediated by a decreased number of B-cell and thus reduced numbers or total absence of immunoglobulins. Moreover, various defects in the cellular immune system such as lymphopenia and impaired T lymphocyte function are described. While the early onset of infections may lead to sepsis and is often a cause of death at a young age, the patient clinical characteristics include autoimmune manifestations such as granulomatosis, enteritis, dermatitis, and vitiligo (58, 59).

With regard to the TCR repertoire, an oligoclonal pattern of the TRBV chain was detected in children with DiGeorge-Syndrom and bone marrow failure (60, 61). In recent studies, oligoclonality could be confirmed using NGS in patients with Wiscott-Aldrich-Syndrom, common variable immune deficiency and X-linked agammaglobulinaemia (58, 59, 62). Additionally, results show a reduced junctional diversity with less nucleotide deletions and insertions in the CDR3 region and a reduced CDR3 length (62). Whether these changes are due to inherited defects or signs of reduced thymic output and subsequent peripheral expansion of T cells is still under research.

### Chronic Infections

Human immunodeficiency virus (HIV), Epstein-Barr virus (EBV), and Cytomegalovirus (CMV) cause lifelong infections in the human host. Their replication is tightly controlled by virus-specific CD8+ T cells (63). In the US, the overall seroprevalence is 50.4% and 66.5% for CMV and EBV, respectively (64, 65). While the role of effector memory T (T_EM_) cells in CMV- and EBV-infected children has recently been evaluated (66), studies on the TCR repertoire in early childhood are lacking. However, in adults, it is known that the CD8+ T cell repertoire in response to CMV infection is highly skewed, due to public TCR that are often dominant within an individual and germline (TCRα chain) or nearly germline-encoded (TCRβ chain) (67). Similar findings have been reported for EBV (11, 68, 69). Further, it has been shown that public recognition of immunodominant EBV epitopes is mainly driven by the TCRα chain (11).

It is already known that HIV-exposed uninfected children have a reduced CD4^+^/CD8^+^+ ratio, as well as CD4+ and CD8+ naïve T cell percentage, but an increased rate of activated CD8+ T cells, and that these abnormalities persist over time (70). Further, their thymic output is reduced, which, together with the lower number of CD4+ cells, is caused by decreased cloning efficiency of their progenitors (71). Newer investigations yielded that human immunodeficiency virus (HIV)-exposed uninfected infants (HEU) has a significantly reduced TCRβ diversity and identifiable clonotypes compared to HIV not-exposed (HU) children and that this reduced diversity is associated with greater numbers of high abundance clonotypes (15).

### Other Chronic Inflammatory Disorders

Celiac disease (CeD) is a chronic inflammatory disease caused by an increase in gut intraepithelial γδT cells due to cereal gluten exposure. Compared to healthy individuals, CeD patients have a more extensive and more diverse γδTCR repertoire, a higher usage of TRGV1 and TRDV3, and different patterns of TCRγ and TCRδ- pairing. However, no CeD-specific γδCDR3 motifs could be detected (72).

Also, for inflammatory bowel disease (IBD), a persistent inflammatory response to gut bacteria, a significant increase in TCR in circulating lymphocytes of IL10/IL10R-deficient patients was observed. The authors found shorter CDR3β length and altered hydrophobicity in T cells but could not find specific TCR clones unique to each patient (73). Further, it has been shown that intestinal TCR repertoires show a lower clonotype diversity and a stronger clonal expansion than those in blood. This loss of diversity is caused by the selective bias of V and J gene usage (74).

For other chronic inflammatory disorders like bronchopulmonary dysplasia (BPD) or idiopathic pulmonary fibrosis (IPF), only little is known about the role of the TCR repertoire. For BPD, it has been shown that the TCR receptor pathway in infants suffering from BPD is significantly down-regulated in comparison to healthy individuals (75). Regarding IPF, it is known that T cells in IPF infants were relatively decreased, but the CD4+ memory T cells, the memory T cells relative to naïve T cells, as well as the CD4/CD8 ratio increased compared to the healthy control group (76).

## Discussion

The development of the human immune system starts within the first weeks of gestation. From about 7 weeks onwards, fetal prothymocytes with rearranged TCR genes occur in the yolk sac and liver. Besides random insertion and deletion of single nucleotides, the genetic rearrangement of the BCR and TCR repertoire is a highly regulated process. In the fetus, the TCR repertoire is limited by clonal restriction and by short CDR3-regions. During ontogeny, these restrictions are gradually released in a controlled manner under the influence of extrauterine antigens. Soon after birth, such changes in the repertoire show a rapid increase, probably dependent on one’s individual microbiome. Ravens et al. could show an increase in γδ T cell subsets in European and African children and a high number of public clonotypes, independent on inherited factors. This increase was reinforced by acute infections but not by vaccination, leading to the hypothesis that only severe immunological challenges leads to a change in the repertoire (77).

In adults, recent research reveals a consistent pattern of oligo- or monoclonal TCR repertoire due to clonal expansion of single TCR clones and a skewed CDR3 length in autoimmune disorders and chronic viral infections. It could be demonstrated that investigations in the TCR repertoire can help to develop new biomarkers. Furthermore, new therapeutic options were established in cancer therapy using engineered T cell subsets (78, 79). Whether these advantages are suitable for clinical application and could be transferred to chronic infections and autoinflammatory diseases is still a topic of research (80).

Until now, there are only a few studies in children focussing on the TCR repertoire and the underlying causes of many chronic disorders in childhood remain unclear. However, chronic disorders in childhood are common and lead to a high burden of disease and often permanent injuries. Some studies in children showed similar findings to the research in the elderly, but findings are not consistent and studies using NGS are still rare. NGS techniques can provide new and high-resolution insights into the TCR repertoire of healthy and diseased individuals. Studies in children using NGS techniques have the potential to unravel the relevance of antigen exposure and the subsequent changes in the TCR repertoire. Understanding the role of fetal/neonatal imprinting and the relevance of specific antigen exposure in early childhood can lead to new diagnostic approaches or even prevention strategies such as vaccination. New studies in infancy and childhood, regarding the age of onset, stage of disease and ongoing therapy are necessary for a better understanding of immune protection on the one hand and dysregulation of the immune response on the other hand.

## Author Contributions

CS and SF planned, structured, and edited the manuscript. SV described the methods and added **Table 1**. All authors searched the literature and integrated all contributions. All authors contributed to the article and approved the submitted version.

## Funding

MZ is supported by Bundesministerium für Bildung und Forschung (BMBF), PRIMAL Consortium grant 01GL1746D. CS is supported by the Universities Giessen and Marburg Lung Center (UGMLC), the German Center for Lung Research (DZL), University Hospital Giessen and Marburg (UKGM) research funding according to article 2, section 3 cooperation agreement, and the Deutsche Forschungsgemeinschaft (DFG)-funded SFB 1021 (C04), KFO 309 (P10), and SK 317/1-1 (Project number 428518790) as well as by the Foundation for Pathobiochemistry and Molecular Diagnostics.

## Conflict of Interest

For CS: Consultancy and research funding, Hycor Biomedical and Thermo Fisher Scientific; Research Funding, Mead Johnson Nutrition (MJN); Consultancy, Bencard Allergie.

The remaining authors declare that the research was conducted in the absence of any commercial or financial relationships that could be construed as a potential conflict of interest.
